# The impact of cardiac magnetic resonance viability assessment on the management of patients with ischaemic heart disease and left ventricular dysfunction

**DOI:** 10.1186/1532-429X-17-S1-P140

**Published:** 2015-02-03

**Authors:** Ben Cole, Hannah Douglas, Chin Munn Soong, Paul Horan, Lana Dixon, Nicola Johnston, Mark Harbinson

**Affiliations:** Cardiology, Royal Victoria Hospital, Belfast, UK; Queens University, Belfast, UK; Antrim Area Hospital, Antrim, UK

## Background

Identifying patients with ischaemic left ventricular dysfunction that would benefit from revascularisation as opposed to medical therapy alone is challenging. Viability assessment has in many centres become a gatekeeper to revascularisation, however its use remains controversial with several prospective randomised trials showing no mortality benefit with this strategy. The aim of this study was to investigate the role of cardiovascular magnetic resonance (CMR) imaging in the decision making process in patients being considered for revascularisation at our institution.

## Methods

All patients referred for CMR viability assessment at a single regional centre were identified retrospectively between January 2011 and March 2013 inclusive. CMR scans were reviewed and segmental viability was determined using the AHA segmental model with viability defined as less than 50% delayed contrast enhancement. Patient records were reviewed to determine the ultimate revascularisation strategy as well as patient outcomes.

## Results

324 consecutive patients were identified, of which 256 were being considered for revascularisation. The remainder were undergoing viability assessment for other reasons, for example valvular heart disease. Of the patients being considered for revascularisation, 38 (14.4%) had preserved left ventricular (LV) systolic function, 33 (17.3%) mild LV dysfunction, 77 (28.4%) moderate LV dysfunction and 108 (39.9%) severe LV dysfunction. Of the patients with severe LV dysfunction 22 subsequently underwent coronary artery bypass grafting (CABG), 30 had percutaneous coronary intervention (PCI) and the remaining 56 patients were managed medically. The patient characteristics are detailed in table 1. All patients undergoing CABG had at least partial viability in the left anterior descending artery territory. In those who underwent PCI, the results of viability testing changed the proposed management strategy in 30%. By the end of the follow period (median, 28 months), death from any cause occurred in 3 patients (14%) in the CABG group, 4 (13%) in the PCI group and 14 (25%) in the medical-therapy group (p=0.32). Hospitalisation for cardiac causes occurred in 1 patient (5%) in the CABG group, 1 (3%) in the PCI group and 28 (50%) in the medical-therapy group (p <0.001). Hospitalisation in the medical therapy group was primarily related to admissions with heart failure (73%).

**Table 1:** Patient characteristics

	CABG (n=22)	PCI (n=30)	Medical therapy (n=56)	P value
Age (yrs)	67.0 ± 9.4	70.5 ± 8.9	69.5 ± 13.0	0.53
Gender (% m)	73	87	86	0.325
LVEF	28.3 ± 4.6	27.4 ± 5.3	25.8 ± 6.0	0.158
LVEDVi (mls/m2)	128.0 ± 29.9	139.8 ± 30.1	139.7 ± 29.5	0.259
LVESVi (mls/m2)	87.3 ± 25.9	101.5 ± 23.6	102.5 ± 25.9	0.049
No. of viable segments	13.1 ± 2.78	12.2 ± 3.0	10.5 ± 3.8	0.005

LVEF- Left Ventricular Ejection Fraction

LVEDVi- Left Ventricular End Diastolic Volume indexed to body surface area

LVESVi- Left Ventricular End Systolic Volume indexed to body surface area

## Conclusions

Patients undergoing surgical revascularisation had significantly less adverse remodelling than those managed with PCI or medical therapy. Patients undergoing CABG with viability prior to revascularisation demonstrated good outcomes, similar to those undergoing PCI. The medical therapy group had a significantly higher number of non-viable segments and outcome was poor. Further randomised controlled trials using CMR assessment of viability may provide evidence that viability assessment is still beneficial prior to revascularisation in patients with ischaemic cardiomyopathy.

## Funding

No external funding.

